# The social neuroscience and the theory of integrative levels

**DOI:** 10.3389/fnint.2015.00054

**Published:** 2015-10-29

**Authors:** Raquel Bello-Morales, José María Delgado-García

**Affiliations:** ^1^Faculty of Medicine, Autonomous University of MadridMadrid, Spain; ^2^Division of Neurosciences, Pablo de Olavide UniversitySeville, Spain

**Keywords:** emergentism, integrative levels, multilevel integrative analysis, reductionism, social neuroscience

## Abstract

The theory of integrative levels provides a general description of the evolution of matter through successive orders of complexity and integration. Along its development, material forms pass through different levels of organization, such as physical, chemical, biological or sociological. The appearance of novel structures and dynamics during this process of development of matter in complex systems has been called emergence. Social neuroscience (SN), an interdisciplinary field that aims to investigate the biological mechanisms that underlie social structures, processes, and behavior and the influences between social and biological levels of organization, has affirmed the necessity for including social context as an essential element to understand the human behavior. To do this, SN proposes a multilevel integrative approach by means of three principles: multiple determinism, nonadditive determinism and reciprocal determinism. These theoretical principles seem to share the basic tenets of the theory of integrative levels but, in this paper, we aim to reveal the differences among both doctrines. First, SN asserts that combination of neural and social variables can produce emergent phenomena that would not be predictable from a neuroscientific or social psychological analysis alone; SN also suggests that to achieve a complete understanding of social structures we should use an integrative analysis that encompasses levels of organization ranging from the genetic level to the social one; finally, SN establishes that there can be mutual influences between biological and social factors in determining behavior, accepting, therefore, a double influence, upward from biology to social level, and downward, from social level to biology. In contrast, following the theory of integrative levels, emergent phenomena are not produced by the combination of variables from two levels, but by the increment of complexity at one level. In addition, the social behavior and structures might be contemplated not as the result of mixing or summing social and biological influences, but as emergent phenomena that should be described with its own laws. Finally, following the integrative levels view, influences upward, from biology to social level, and downward, from social level to biology, might not be equivalent, since the bottom-up processes are emergent and the downward causation (DC) is not.

## Introduction

It is an old observation that living systems are structured in hierarchical levels of organization, in which the entities of one level are compounded into new entities at the next higher level (O’Connor and Wong, 2012). This observation has given rise to diverse theoretical constructs, among them the concept of integrative levels, an idea that underwent an interesting debate from the second half of the twentieth century (Gerard and Emerson, 1945; Needham, 1945, 1986; Novikoff, 1945; von Bertalanffy, 1950; Feibleman, 1954; Gerard, 1957; Mayr, 1982; Aronson, 1984). Physical, chemical, biological, mental or sociological spheres have been proposed as primary levels of organization (Emmeche et al., 1997, 2000). Several sub-levels, such as molecules, organelles, cells, tissues, organs, physiological systems, neural circuits and information processing systems, psychological faculties and functions, memories… and so on, have also been proposed (Kirmayer and Gold, 2011), and several mesoforms can also exist (Novikoff, 1945). It has been asserted that the development of matter through its different forms of motion gives rise to new integrative levels of complexity, whose emergent properties appear only when the constituent elements of the lower level are combined in the higher level (Novikoff, 1945). Concepts such as emergence, nonlinearity, attractors and self-organization have contributed to transform mechanistic and reductionist views (Goldstein, 1999; Mazzocchi, 2008), and current research in self-organizing non-linear dynamical systems has impelled the scientific study of emergence, giving rise to its final devitalization or demystification (Emmeche et al., 2000).

According to the concept of emergence, the qualities of complex systems cannot be derived from the characteristics of the components alone: the whole is greater than the sum of its parts, as classically expressed (Goldstein, 1999; Van Regenmortel, 2004; Singer, 2007; Findlay and Thagard, 2012). Nevertheless, it has been argued that it is not proper enough to say that the whole is more than the sum of its parts: indeed, the properties of the parts cannot be understood except in their context in the whole (Lewontin, 1993). On the other hand, the different levels of organization are not completely delimited from each other (Novikoff, 1945; Emmeche et al., 2000), which does not mean that levels do not possess any ontological status. Indeed, integrative levels of organization have been considered not only as levels of description. On the contrary, an ontology of at least four primary levels, physical-chemical, biological, psychological and sociological, have been suggested (Emmeche et al., 1997). Diverse and divergent points of view have been embraced under the emergentist approach. Teilhard de Chardin, for instance, incorporated in his philosophy the concept of emergent levels with a teleological character, arguing that at each further degree of combination, something that is irreducible to isolated elements emerges in a new order, and incorporating a mystical content according to which the evolution progresses towards the consciousness (Teilhard de Chardin, 2008; Blitz, 2010).

## Levels of Organization and Emergence

The development of matter in the universe has been attributed to the combination of the fundamental laws and a contribution from historical accidents, and although to reduce one level to the lower one—together with historical contingence—is possible in principle, it does not by itself allow to understand the world: at each level of organization new regularities emerge that should be studied for themselves, and new phenomena should be appreciated and valued at their own level. That is, larger-scale phenomena have their own dynamics and therefore, macro-level processes require their own language of description (Gell-Mann, 2001; Kirmayer and Gold, 2011). In words of Anderson (1972), the ability to reduce everything to simple fundamental laws does not imply the ability to start from those laws and reconstruct the universe. To define emergence is not a trivial issue and a considerable amount of different interpretations has been generated around this question since J. S. Mill and later the British Emergentism started to address it (Goldstein, 1999; O’Connor and Wong, 2012). However, it is widely accepted that emergent properties are novel and unpredictable from knowledge of their lower levels and they cannot be explained or mechanistically reduced in terms of their underlying properties (Kim, 1999). Emmeche et al. (1997, 2000) considers that emergent levels are inclusive, that is, they do not violate any physical laws and in addition, they permit the local existence of different ontologies. What is more, emergent levels not only violate any physical law, but they also might be obeying fundamental physical laws throughout the successive levels: for example, the general tendency of events towards “disorder” is observable in any level of organization, although in organic development and evolution, a transition towards states of higher complexity and differentiation also occurs (von Bertalanffy, 1950). Certain characteristics of live matter, such as those related to the basic necessities of living organisms—for instance survival, security and exploration or search—might also function along the entire development of life processes. In other words, while certain physical or biological laws are only applicable to certain levels of organization, other fundamental laws are applicable to the matter along its entire development.

Emergence is, therefore, the arising of a higher-level phenomenon from a lower system level, and hence, it is an upward process. This upward causation is characterized by a causal process leading from the lower level entities to the higher level ones, so that the lower level can be considered as the cause and the higher level as the effect (Emmeche et al., 2000). However, emergent phenomena can feedback to lower levels, causing changes through a process called downward causation (DC; Campbel, 1974; Mayr, 1982; Emmeche et al., 2000), in which wholes can affect properties of components at lower levels.

The existence of different levels of organization does not imply necessarily the existence of different substances, since there is an unbroken continuity of material constitution along the development of the different levels of complexity; all of them are just part of a single (material) world (Kirmayer and Gold, 2011). Strong DC, based on a constitutive irreductionism, considers that higher level entities acquire a substantial existence qualitatively different from lower level entities, but other forms of DC maintain that the higher levels does not add any substance to the entities of the lower level and that, in addition, constitutive irreductionism and substantial realism of levels support an unscientific principle that infringe the inclusivity of levels (Emmeche et al., 2000). In short, the integrative levels describe a unique material world with many hierarchical levels of complexity. In relation to the mind-body question, the mind does not involve a different substance but, ontologically, originates from the interactions of brain, body and environment (Kirmayer and Gold, 2011). Asserting that consciousness and mental states can be reduced to chemical reactions taking place in the brain has been considered as the most extreme manifestation of the reductionist view (Van Regenmortel, 2004). The reductionist approach to study the behavior of living beings—or of their *alter ego*, the brain—may provoke the absurd feeling that the more precize the detail that is discovered, the less acceptable it is to explain something complex (Delgado-García, 2011). Perhaps experimental neuroscientists are excessively focused toward the inside of the neuron in search of the molecules that make possible learning or social interactions and therefore, in the reasonable curiosity to find at the lower integration level the explanation for a given functional process, we might be at risk of forgetting the search for the origin of emergent properties (Delgado García, 2015). Zaki and Ochsner (2012) have also remarked that important problems may emerge when researchers rely on overly simplified models of complex psychological phenomena, especially when they try to “scale up” or extrapolate data from such models into general conclusions. They consider that complex cognitive phenomena are difficult, if not impossible, to describe through data about their building blocks alone, since these phenomena are greater than the sum of their parts.

Other positions sustain that humans are embedded in a complex network in which society, culture and history are determinant factors, and although obviously the mind depends on the brain, it cannot be isolable within it. This position is not dualistic, since it does not admit the existence of a different immaterial substance underlying these mental phenomena, but it seeks to reject the old mechanical materialism that pretends to reduce higher order phenomena to lower ones, a kind of reductionism in which mind is an epiphenomenon of the brain, as it was considered by the mechanical materialists of the nineteenth century (Rose, 2012).

## Emergence of Human Consciousness and Behavior

The human brain, the most complex known structure, is considered as a complex system in which mental states emerge from the interaction between multiple physical and functional levels (Bassett and Gazzaniga, 2011). But the human brain is also embedded in the levels that surround it and therefore, the nervous system, the body and the environment have been contemplated as dynamical systems coupled with one another on multiple levels, that is, mutually embedded systems (Thompson and Varela, 2001). Moreover, in the brain, emergent phenomena can feedback to lower levels (Bassett and Gazzaniga, 2011; Delgado-García, 2011), causing changes through DC. The physiological effects produced by certain psychological states have been proposed as an example of DC (Campbell and Bickhard, 2011).

Vygotsky proposed that the mental functions along human cultural development appear first on the social level, and later, on the individual level. The higher functions originate, according to him, as actual relations between human individuals. The transformation of interpersonal processes into intrapersonal ones would be, therefore, the result of a long sequence of developmental events and the internalization of socially rooted and historically developed activities is the distinctive feature of human psychology, the basis of the qualitative leap from animal to human consciousness (Vygotsky, 1978, 1986). The transition of elementary to higher mental functions is—according to Vygotsky—mediated by psychological tools, which have a semiotic nature: language, systems for counting, writing, algebraic symbol systems, and so on. Vygotsky’s description of the origin of higher mental functions is greatly based on these forms of mediation (Wertsch, 1985). When using the term “social”, Vygotsky was referring to “face to face”, dyadic or small group communicative processes: interpsychological processes. Nevertheless, he recognized another level of social phenomena as well: “social institutional” processes, as termed by Wertsch. Interpsychological processes are not reducible to individual psychological processes, which would constitute a form of psychological reductionism and likewise, societal processes cannot be reduced to interpsychological processes, which Wertsch considers another form of reductionism.

## Social Neuroscience

Three decades ago, the social neuroscience (SN) started to emerge as a new field, undergoing since then an important expansion and growth. This new interdisciplinary field which aims to investigate the biological—neural, hormonal, cellular, and genetic—mechanisms that underlie social structures, processes, and behavior and the influences between social and biological levels of organization, has been considered useful in several aspects of the social life, such as education, health or public policy (Cacioppo and Decety, 2011) and has received considerable attention from the media and the general public (Stanley and Adolphs, 2013). This new focus pervaded cognitive neuroscience giving rise to the social cognitive neuroscience approach, which seeks to understand phenomena in terms of interactions between social, cognitive and neural levels of analysis (Ochsner and Lieberman, 2001). SN has declared the necessity for including social context as an essential element to understand the human behavior and it has tried to overcome the purely biological stance of some viewpoints introducing in its conceptual framework its doctrine of multilevel analysis (Cacioppo and Berntson, 1992).

SN has given rise to abundant scientific works, many of them in the realm of neuroendocrinology. It has been established that certain neuropeptides and steroid hormones have a sex-specific effect on animal social behavior (Donaldson and Young, 2008; Insel, 2010; Bos et al., 2012; Goodson, 2013), modulating social behavior and social cognition. Oxytocin and arginine vasopressin (AVP)—so called “social neuropeptides”—are important modulators of diverse social behaviors, including attachment, social recognition and aggression (Veenema and Neumann, 2008; Heinrichs et al., 2009; Ebstein et al., 2012; Goodson, 2013; Kelly and Goodson, 2014; Lieberwirth and Wang, 2014). Neuropeptides have also been involved in the etiology of autism spectrum disorders (Insel et al., 1999; Bartz and Hollander, 2008; Green and Hollander, 2010; Meyer-Lindenberg et al., 2011).

Oxytocin has been involved in the modulation of a wide variety of behaviors in mammals, and humans in particular, such as aggression, affiliation or social memory (Young and Wang, 2004; Lee et al., 2009; Ross and Young, 2009), and its role in social cognition in humans has been the focus of extensive research in recent years (Guastella and MacLeod, 2012). Thus, the findings of SN in animal behavior have led to the search for its human analoges. For instance, it has been reported that oxytocin increases trust (Kosfeld et al., 2005) and generosity (Zak et al., 2007) in males, although, interestingly, oxytocin does not seem to uniformly facilitate trust and pro-social behavior in humans, since it may also impede trust and pro-social behavior depending both on diagnosis and on chronic interpersonal insecurities combined with situational factors (Bartz et al., 2011). Oxytocin administration also increases ratings of trustworthiness and attractiveness of male and female targets, suggesting that higher levels of this nonapeptide may enhance affiliative behavior towards unfamiliar others (Theodoridou et al., 2009). In an acute administration study, fathers were more stimulating of their child’s exploration than in the placebo condition, and they tended to show less hostility (Naber et al., 2010). Also, oxytocin significantly increased positive communication behavior in relation to negative behavior during a couple conflict discussion and significantly reduced salivary cortisol levels after the conflict, when compared with placebo (Ditzen et al., 2009). Oxytocin also seems to enhance the buffering effect of social support on stress responsiveness (Heinrichs et al., 2003). Furthermore, oxytocin has also been suggested to be a key element in social motivation (Gordon et al., 2011), parochial altruism (De Dreu et al., 2010) and cooperation within groups (De Dreu, 2012). The pronounced reduction in activation and amygdala–midbrain connectivity in males receiving oxytocin revealed a marked impact of this neuropeptide on amygdala reactivity and brainstem interactions in humans suggesting a neural mechanism for its effects in social cognition (Kirsch et al., 2005). Regarding memory, intranasal administration of oxytocin specifically improved recognition memory for faces, but not for non-social objects (Rimmele et al., 2009). On the other hand, oxytocin has been reported to promote human ethnocentrism—the tendency to view one’s group as centrally important and superior to other groups—(De Dreu et al., 2011) as well as increasing envy and *schadenfreude* (gloating; Shamay-Tsoory et al., 2009). In addition, a role has been suggested for this neuropeptide in group-serving dishonesty, a finding that has been claimed to support a functional approach to morality (Shalvi and De Dreu, 2014).

Regarding AVP, a regulator role has been reported for this nonapeptide in maternal (Bosch and Neumann, 2010; Bosch, 2013) and intermale (Ferris and Potegal, 1988) aggression in rodents. AVP seems to modulate intermale aggression eliciting region-specific effects (Veenema et al., 2010), that is, by either promoting or inhibiting aggression depending on the brain region into which it is released. Although similar neurochemical and neuroanatomical pathways are activated in mice and humans, it has been argued that the enormous differences in biology and social structure make it unlikely for mouse and human aggression to be classified into homologous categories. Furthermore, relating behavioral, neurobiological and molecular mechanisms of aggression in non-human animals to the human condition is not easy (Nelson and Trainor, 2007). AVP effects appear to be sex-specific, promoting agonistic and affiliative types of responses toward same-sex faces in men and women, respectively (Thompson et al., 2006). Other studies have also suggested that both behavioral and neural responses to intranasal oxytocin and AVP are highly sexually differentiated (Rilling et al., 2014). Intranasal AVP administration also enhances the encoding of emotional facial expressions (Guastella et al., 2010) and the cognition for sexual stimuli (Guastella et al., 2011) in human males. A role for AVP in enhancing aggressive behavior in personality-disordered individuals has been also suggested (Coccaro et al., 1998).

It is considered that the amygdala plays a critical role in the affective and motivational drive to respond aggressively to social provocation, while the orbitofrontal cortex is thought to be a self-regulatory region that inhibits aggressive impulses (Mehta et al., 2013). Recently, it has been shown in behaving mice and rabbits that the prefrontal cortex plays a restrictive role in the release of spontaneous or recently acquired (i.e., learned) behaviors (Jurado-Parras et al., 2012; Leal-Campanario et al., 2013). Testosterone has been associated to affiliative behavior, stress response and social aggression. The effect of testosterone on aggression has been explained by a reduction in activity in the medial orbitofrontal cortex (Mehta and Beer, 2010). Although elevated testosterone levels have been positively associated with aggressive behavior in animals, it has been claimed that situationally induced fluctuations in testosterone levels are more relevant to human aggression than stable levels of testosterone (Mehta et al., 2013; Carre and Olmstead, 2015).

However, although there is some evidence suggesting a role for testosterone in aggression, the results are controversial and according to other authors, conflicting and inconclusive (Eisenegger et al., 2011a). Thus, sublingual administration of testosterone in women caused a substantial increase in fair bargaining behavior, thereby reducing bargaining conflicts and increasing the efficiency of social interactions. However, subjects who believed that they had received testosterone—regardless of whether they actually received it or not—behaved much more unfairly than those who believed that they had been treated with placebo. Thus, folk beliefs about the effects of testosterone—that testosterone induces antisocial, egoistic, or even aggressive human behaviors—seem to generate that unfairly behavior (Eisenegger et al., 2010), which highlights the relevance of psychological factors on the results obtained from neuroendocrinological studies. Earlier studies had also suggested that testosterone causes expectations, rather than inducing an actual increase in aggressiveness (Björkqvist et al., 1994). These results have been criticized with the argument that testosterone increases reactive aggression in men, but not in women, and that social environment can moderate testosterone-behavior associations (Josephs et al., 2011). In this controversy, Eisenegger et al. have questioned the hypothesis that testosterone causes aggressive motivation in men, arguing that no sound evidence for a gender-specific effect of testosterone on aggressive motivation exists. Instead, they have suggested that testosterone drives a more general range of motivated behaviors, often subsumed under the concept of dominance behavior, that is, the motivation to achieve or maintain a high social status, which appears to be non-aggressively achieved in primates (Eisenegger et al., 2011a). Caution when interpreting data from correlational studies is a common demand. For instance, data from salivary testosterone levels in male and female prisoners showed that inmates who had committed personal crimes of sex and violence had higher testosterone levels than inmates who had committed property crimes of burglary, theft, and drugs, indicating a positive correlation between endogenous testosterone levels and the exhibition of aggressive, egoistic and anti-social behavior (Dabbs et al., 1995; Dabbs and Hargrove, 1997). However it has been argued that causality in these studies remains unclear, since the higher levels of aggression might well have caused the higher testosterone levels, leaving open the question about the role of testosterone in this behavior (Eisenegger et al., 2011b).

Other issues such as social decision making (Lee and Harris, 2013), morality (Sobhani and Bechara, 2011; Young and Dungan, 2012), reputation (Izuma, 2012) or empathy (Singer and Lamm, 2009; Decety and Svetlova, 2012) are being also addressed by SN. The SN approach on empathy research has experienced a significant growth in the last decade. Empathy has been defined as the experience of vicariously feeling what another person is feeling without confounding the feeling with one’s own direct experience (McCall and Singer, 2013). Nevertheless, the term empathy has been applied to many distinct phenomena, such as knowing another person’s internal state, coming to feel as another person feels, imagining how another is thinking and feeling or feeling distress at witnessing another person’s suffering (Batson, 2009). Others consider empathy as a set of related but distinct processes through which “perceivers” (individuals focusing on another person’s internal states) relate to “targets” (individuals who are the focus of perceivers’ attention). These processes are grouped into three broad classes: experience sharing—vicariously sharing targets’ internal states—, mentalizing—explicitly considering (and perhaps understanding) targets’ states and their sources—and prosocial concern—expressing motivation to improve targets’ experiences—(Zaki and Ochsner, 2012). Zaki and Ochsner have argued that relying too heavily on highly simplified models may introduce interpretational confusion into existing models of empathy. In addition, in the absence of brain—behavior relationships, conclusions about the role of certain brain regions in experience—sharing tasks may require reverse inference. Therefore, many findings in this field depend for their accurate interpretation on the incorporation of behavior into the neuroscience of empathy. To avoid these and other pitfalls, the above authors propose a few simple changes on the focus, many of which are already gaining force within a new generation of empathy studies (Zaki and Ochsner, 2012).

Oxytocin and AVP are being considered as promising targets for clinical treatment approaches for social dysfunctions such as autism, social anxiety disorder, borderline personality disorder and schizophrenia (Meyer-Lindenberg et al., 2011). Nevertheless, the acute administration techniques used in the studies on humans have methodological problems: for instance, it is not clear what proportion of hormone reaches the brain or what is the exact relationship between the peripheral measures and the central levels. The uncertainties surrounding the intranasal administration of oxytocin invite to be cautious when interpreting the data, and some authors have raised several questions to be resolved. Do intranasal administrated hormones reach the brain? Do they reach its receptors? What constitutes a sufficient dose to ensure a behavioral effect? How do the exogenous hormones interact with other substances? In this sense, Churchland and Winkielman (2012) have argued that it is unlikely that any widely acting hormone or neurotransmitter will modulate complex mental processes specific to social cognition. Instead, they propose explanations in terms of more general mechanisms. For instance, higher-order social-cognitive effects observed in humans might be due to the anxiolytic effect triggered by oxytocin. For these authors, it is doubtful that oxytocin directly influences complex human social cognition.

Besides social behavior, it has been also suggested that hormones can modulate moral behavior through their effects on the brain, in addition to other factors such as genetic polymorphisms, which can predispose to aggression and violence. According to this reductionist view, the “moral brain” would be a large functional network including cortical (frontal, temporal and cingulated cortices) and subcortical (amygdala, hippocampus and basal ganglia) anatomical structures and accordingly, abnormal moral behavior might arise from both functional and structural brain abnormalities that should be diagnosed and treated (Fumagalli and Priori, 2012). It has even been proposed a genetic basis for political beliefs and in general, for numerous aspects of the human social behavior. For instance, it has reported that the serotonin transporter SLC6A4, the monoamine oxidase A and the dopamine receptor D2 genes function might have an effect on voting preferences (Ebstein et al., 2010).

### Downward Causation in Social Neuroscience

SN aims to study not only the biological mechanisms that underlie social structures and behavior, but also the effects that social level of organization exerts on the human physiology and behavior (Cacioppo et al., 2011; Eisenberger, 2012, 2013). For instance, it has been observed the effect of social isolation in humans (Cacioppo et al., 2011) or the effect of stressful environment on brain and behavior, suggesting that early life stress induces structural changes—in particular, an increase in the volume of the amygdala and a decrease in certain sectors of the prefrontal cortex and hippocampus—(Davidson and McEwen, 2012). In this context, epigenetic modifications have been proposed as a possible mechanism involved in early positive and negative social experiences, suggesting that early social life experiences might induce epigenetic DNA changes in the developing brain with lifelong impacts (Hoffmann and Spengler, 2014). Epigenetics may be a mechanism through which the social environment becomes embedded at a biological level (Champagne, 2010).

Epigenetics has been considered as the climax in the process of “socialization” of biological and neurobiological concepts as well as the last frontier in the development of a narrative about the sociality of the brain, and the discovery of a mechanism mediating between environmental exposures, gene expression and neuronal development, that might confirm many of the insights of SN research since the 1990s (Meloni, 2014). An emblematic study showed that maternal behavior in the rat can alter the hippocampal glucocorticoid receptor (GR) expression in the offspring, which concomitantly alters the hypothalamic-pituitary-adrenal (HPA) axis and the stress responsiveness of these animals. Maternal behavior increases GR expression in the offspring via increased hippocampal serotonergic tone accompanied by increased histone acetyltransferase activity, histone acetylation and DNA demethylation. In addition, cross-fostering studies showed that pups exhibit the behaviors of foster parents (Weaver et al., 2004). This study demonstrates that a specific epigenetic state of a gene can be established through early life experiences (Weaver, 2007). Regarding human studies, a recent one examined epigenetic differences between a neuron-specific GR promoter from a postmortem hippocampus obtained from suicide victims with a history of childhood abuse, and the same promoter from postmortem hippocampus obtained from either suicide victims with no childhood abuse or from controls. Hypermethylation of the GR gene was found among suicide victims with a history of abuse in childhood, but not among controls or suicide victims with a negative history of childhood abuse (McGowan et al., 2009). These results are consistent with evidence from other studies which suggests that suicide has a developmental origin. They are also consistent with the hypothesis that early life events can modify the epigenetic state of genomic regions whose expression might contribute to individual differences in the risk of psychopathology (McGowan and Szyf, 2010). The ways in which social structures and socio-economic differences literally get under the skin (and in the brain), influencing the human physiology, has always been relevant for sociological theory (Meloni, 2014). In this context, we contemplate epigenetic changes, generated from the social level onto the biological one, as a paradigm of DC. Top-down social processes affecting biology might take place mostly via epigenetic mechanisms.

### From Animal Models to Human Research

Animal models have been foundational to the SN (Cacioppo and Decety, 2011) and some consider that animal research will continue to be important for resolving the critical questions driving current work in humans (McCall and Singer, 2012). Some studies suggest that the molecular pathways and neural networks mediating social behavior are relatively conserved throughout evolution in mammals (Weitekamp and Hofmann, 2014). In this context, the findings of neuroendocrinology on animal behavior have promoted the search for its analoges in humans. Nevertheless, although it is usual to look for “animal models” of human disorders or to assume that findings in animals can be extrapolated directly to humans, the translation from animal models to human research might be built with careful consideration of species differences, since although some of the principles may be conserved, the details for social organization need to be explored for each species, recognizing the importance of diversity in the neural mechanisms for social cognition (Insel, 2010). Regarding the studies with great apes, experimental research has revealed important methodological difficulties. A major methodological challenge of experimental research in primate cognition is to design novel problems for individuals and ecologically valid for the species. Also, the findings of experimental research must be comparable among different populations of individuals of the same species. Nevertheless, primates are confronted with cognitive problems that are not well matched with the cognitive abilities that have evolved in their natural environments, since much of the research in primate cognition is conducted with captive populations in the laboratory and enculturated apes. Thus, the generalization of an experiment with laboratory animals to conspecifics in the wild may be inaccurate, because captive individuals in their human-designed environments might have developed a unique set of cognitive skills (Tomasello and Call, 2011). Coinciding with this opinion, other authors have argued that behavioral studies designed to understand the neural bases of behavior should use realistic social situations where animals interact as they would in their natural habitats (Insel and Fernald, 2004). Insel and Fernald (2004) have alerted against the tendency to use simple behavioral assays to investigate complex behavior, remarking that such assays can lead to misinterpretation of results. Therefore, to use ethologically relevant tasks is necessary to avoid anomalous results. The control of visual processing over vomeronasal signals or the prevalence of cortical networks over neuropeptide signals from the hypothalamus questions the validity of the extrapolation of the results obtained in rodents to higher primates or humans. Novikoff also argued that positions that confer on animals, even higher primates, certain human attributes might fall in an erroneous anthropomorphism if they transfer the higher level (social) into the lower level (biological), overlooking the fundamental qualitative differences between them and forgetting that only human society might be considered truly sociological (Novikoff, 1945).

### The Doctrine of Multilevel Analysis

It has been argued that SN can provide a full explanation of social behavior only through a multilevel integrative approach. This doctrine of multilevel analysis is essentially based on three principles (Cacioppo and Berntson, 1992; Cacioppo et al., 2010). The first one is the principle of multiple determinism according to which an event at one level of organization can have multiple antecedents within or across levels of organization. More generally, the principle of multiple determinism means that we are unlikely to achieve a complete understanding of social behavior if we limit analyses to any single level of organization. It has been proposed as an example of this principle the role of the endogenous opioid receptor system in drug use. It has been reported both the contribution of individual differences in the endogenous opioid receptor system in drug use and the important role of social context, suggesting that both factors are involved and according to the authors, the understanding of drug abuse is incomplete if either level is excluded (Cacioppo and Decety, 2011). The second principle is the nonadditive determinism, which specifies that properties of the whole are not always readily predictable from the properties of the parts. The third principle, reciprocal determinism, specifies that there can be mutual influences between biological and social factors in determining behavior. For example, not only the level of testosterone in nonhuman male primates has been shown to promote sexual behavior, but the availability of receptive females influences the level of testosterone in nonhuman primates. According to this view, the understanding of mind and behavior could be enhanced by an integrative analysis that encompasses levels of organization ranging from genes to cultures.

The three principles in which the doctrine of multilevel integrative approach is based seem to correspond to the principles classically used to describe the integrative levels of organization of the matter and the concept of emergence (Novikoff, 1945; Feibleman, 1954; Gerard, 1957; Mayr, 1982; Needham, 1986; Emmeche et al., 1997, 2000). The principle of nonadditive determinism, which specifies that properties of the whole are not always readily predictable from the properties of the parts, corresponds to the concept of emergentism, according to which, the collective behavior of a complex system generates higher-level properties not directly predictable from the lower-level behavior of the system. The principle of reciprocal determinism, according to which there can be mutual influences between biological and social factors in determining behavior, relates to inter-level causation, and is consistent with the concepts of upward and DC (Campbel, 1974). The principle of multiple determinism, according to which an event at one level of organization can have multiple antecedents within or across levels, is consistent with the nature of the interdependence among the different stages of material development. Multiple relations of interdependence can take place inside the levels (intra-levels) or between the levels (inter-levels).

## Conceptual Divergences Between SN and the Theory of Integrative Levels

Despite its recognition of the importance of the social influences on brain and behavior, SN has been criticized for its tendency to focus on lower-level biological phenomena, a fact that has been considered as an important limitation of current work in this discipline (Kirmayer and Gold, 2011). SN asserts that combination of neural and social variables can produce emergent phenomena that would not be predictable from a neuroscientific or social psychological analysis alone (Cacioppo et al., 2010). Nevertheless, it might be considered another point of view, suggesting that emergent phenomena are not produced by the *combination* of variables from *two* levels, but by the increment of complexity at *one* level. In this sense, emergence has been defined as the arising of novel and coherent structures, patterns, and properties during the process of self-organization in complex systems (Goldstein, 1999). According to this view, emergence describes the passage between levels, and thus emergent properties would not arise from the combination of two levels but from the collective behavior of complex systems (Emmeche et al., 1997). Novikoff (1945) also considered laws as unique for each level, not as combinations of laws from other ones.

Second, SN suggest that to achieve a complete understanding of social behavior we cannot limit analyses to any single level of organization, but instead, we should use an integrative analysis that encompasses levels of organization ranging from the genetic level to the social one (Cacioppo et al., 2010). Nevertheless, to understand one level, the theory of integrative levels suggest to use methods of research and analysis appropriate to that particular level, not to encompass or to “mix” all the levels of organization (Novikoff, 1945; Feibleman, 1954; Needham, 1986). It has been alleged that in the human behavior both, social and biological influences operate (Cacioppo et al., 2010). However, to propose that several factors operate does not explain what “determines” human behavior. It could be argued that it is the “sum” of those factors, but it would not agree with the principle of nonadditive determinism, which recognizes that the whole is not the sum of the parts. In contrast, the human behavior might be contemplated—following the theory of integrative levels—not as the result of mixing or summing social and biological influences, but as an emergent phenomenon that might be described with its own laws.

The principle of reciprocal determinism establishes that there can be mutual influences between biological and social factors in determining behavior. Therefore, it accepts a double influence, upward from biology to social behavior, and downward, from social level to biology. However, we argue that those two processes bottom-up and up-down may not be comparable, since the first one, unlike the second one, is emergent, and an emergent process cannot be predicted, *a priori*, from the laws of the lower levels (Novikoff, 1945; Emmeche et al., 1997, 2000; Kim, 1999; O’Connor and Wong, 2012).

SN aims to find the biological mechanisms that underlie *social structures* ranging from dyads and families to groups and cultures (Cacioppo et al., 2010; Cacioppo and Decety, 2011). Comprising all those levels—from dyads to cultures—under the same “social structures” concept, might be imprecise, given the huge ontological distance that separates dyads from cultures or others large human organizational structures, a distance that might even imply a different level of organization. Indeed, while a dyad involves an interpsychological process related to an interpsychological level, a large human organizational structure might correspond to a “social institutional” process, related to a “societal” level (Wertsch, 1985). In addition, some consider that the data from acute administration neuroendocrinological studies provide a promising picture of the central hormonal influence on human *social life* (McCall and Singer, 2012). It has also been claimed that understanding the neurobiology and neurogenetics of social cognition and behavior may have important implications for *society* (Donaldson and Young, 2008). Nevertheless, the vast majority of the neuroendocrinological studies refers to anonymous interactions and interpsychological processes, “face to face”, dyadic or small group communicative processes, not to the level of social phenomena or “social institutional” processes involving large social groups. Therefore, the extent to which neuroendocrinology affects *social structures*, *social life* or *society* should be more rigorously delimited. Even though hormonal studies lacked methodological problems, they would only be related to a very specific level of organization, the one which relates to dyadic or small group processes, but societal processes cannot be reduced to interpsychological processes. For instance, we cannot use the same paradigms to explain aggressiveness between two individuals—a dyadic process—and aggressiveness between two social groups, classes or cultures—societal processes (following Wertsch terminology). Finally, we consider that the vague or imprecise use of the terms *social structures*, *social life* or *society*, without the delimitation of their exact scope, might lead to confusion, especially when the results of SN reach the general public.

## Conclusions

The theory of the integrative levels (Novikoff, 1945; Feibleman, 1954; Aronson, 1984; Needham, 1986) describes the evolution of the matter, from the subatomic dimensions to the social world, claiming that the increment in complexity is the result of forces, different in each level, which can only be properly described by laws which are unique for each level. Emergence refers, therefore, to the arising of novel structures and properties during the process of self-organization in complex systems. It is not possible to understand the origins of the higher levels without an understanding of the lower level phenomena as well. Thus, integrative levels of organization allow us to know the evolution from the inanimate to the animate and social worlds (Lobo, 2008). Nevertheless, knowledge of the lower levels does not make possible to predict, *a priori*, what will occur at a higher level. Therefore, the laws unique to a certain level can only be found out using approaches appropriate to that particular level. When suggesting some basic rules of explanation, Feibleman (1954) also argued that every organization must be explained finally on its own level, adding that organizations must be considered as belonging in some peculiar way to its highest level. According to Goldstein, it is necessary to appeal to emergence when the configuration of the components of a complex system offers a more explanatory insight into the dynamics of the system than do explanations based on the parts alone. Emergence is thus a way to describe the need to go to the macro level and its unique dynamics, laws, and properties in order to explain phenomena more adequately (Goldstein, 1999).

In its basic theoretical formulation, SN has introduced the basic principles that constitute the model of integrative levels, encompassing them under the so called doctrine of multilevel analysis (Cacioppo and Berntson, 1992; Cacioppo and Decety, 2011). In this article, we have aimed to expose the differences between both doctrines, suggesting that the multilevel integrative approach used by SN has significant discrepancies with the classical principles of integrative analysis—although apparently it has common arguments.

First, SN asserts that combination of neural and social variables can produce emergent phenomena that would not be predictable from a neuroscientific or social psychological analysis alone (Cacioppo et al., 2010). Nevertheless, following the classical integrative analysis, emergent phenomena are not produced by the *combination* of variables from *two* levels, but by the increment of complexity at *one* level. In addition, SN suggests that to achieve a complete understanding of social structures we should use an integrative analysis that encompasses levels of organization ranging from the genetic level to the social one (Cacioppo et al., 2010). Perhaps, the concept “social structure” should be more exactly delimited. In addition, the theorists of integrative analysis have suggested that organizations must be explained on its own level, that is, its highest level of organization (Feibleman, 1954). Likewise, the social behavior and structures may be considered not as the result of mixing or summing social and biological influences, but as emergent phenomena that might be described with its own laws. SN establishes that there can be mutual influences between biological and social factors in determining behavior. Therefore, it accepts a double influence, upward from biology to social level, and downward, from social level to biology. However, those two influences might not be situated in the same explanatory nor causal level, because those two processes are not equivalent: the bottom-up processes are emergent and the DC is not.

Finally, SN considers that the data from neuroendocrinological studies provide an adequate explanation of the central hormonal influence on human social life. However, several observations might be indicated here. First, extrapolation from animal studies might be erroneously formulated if simple behavioral assays are used to investigate complex behaviors. In addition, the vast majority of the neuroendocrinological human studies are addressing interpsychological processes, “face to face”, dyadic or small group communicative processes, not social phenomena or “social institutional” processes involving large social groups. Even though hormonal studies lacked methodological problems, they would only be related to that specific level of organization, not to the societal one. If we consider that social events have their own dynamics, creating a direct or indirect bridge between these events and the amount of a certain biological factor may lead us to a simple and flawed position.

## Conflict of Interest Statement

The authors declare that the research was conducted in the absence of any commercial or financial relationships that could be construed as a potential conflict of interest.
